# Ready-to-Eat Innovative Legumes Snack: The Influence of Nutritional Ingredients and Labelling Claims in Italian Consumers’ Choice and Willingness-to-Pay

**DOI:** 10.3390/nu15071799

**Published:** 2023-04-06

**Authors:** Alessandro Petrontino, Michel Frem, Vincenzo Fucilli, Antonella Labbate, Emanuela Tria, Francesco Bozzo

**Affiliations:** 1 Department of Soil, Plant and Food Sciences, University of Bari Aldo Moro, Via Amendola 165/A, 70126 Bari, Italy; 2 Sinagri s.r.l., Spin Off of the University of Bari-Aldo Moro, Via Amendola 165/A, 70126 Bari, Italy

**Keywords:** choice experiment, healthy eating, hedonic price, legumes snack choice determinants, legumes snack quality and preferences

## Abstract

The global offer of legume-based snacks has sharply increased in recent years. However, to date, few studies have focused on the relationship between product supply and demand concerning the importance of attributes of such innovative foods. In this research, we identified the key internal and external determinants that affect legumes snack (LS) price and choice by Italian industries and consumers, respectively. In parallel, we investigated their preferences and perceptions towards these foods. We used the hedonic price model (HPM) and the discrete choice experiment (DCE) approach for these purposes, respectively. HPM revealed that the monetary value of LS was determined to greater significance by the: (i) size of the package; (ii) presence of rice, presence of lentils; (iii) presence of the nutritional information; and (iv) the discount shops as site of purchase. DCE revealed that the: (i) origin certification, (ii) recyclability of the package, and (iii) use of extra virgin olive oil of LS provided Italian consumers a high utility, for which they were willing to pay an average price premium of EUR 3.85, 3.64, and 1.87, respectively. On the contrary, the sunflower oil induced a decrease in their function utility. As such, this paper contributes to define potent market-segmentation strategies and to deliver effective private and public nutrition interventions for healthy eating.

## 1. Introduction

### 1.1. Broad Context

In many countries around the world, improving food nutrition and, consequently, eating healthy is of greater importance on the political agenda. This increased emphasis on health has created a market for food products promoted as healthy or associated with wellness [1]. As such, food manufacturers reframe their labelling processed foods for healthier claims [2], affecting the consumers’ perceptions of the healthiness of processed packed foods [3] and helping them to choose among these products, for which nutritional ingredients and labelling information should be unambiguous and comprehensible [4].

In this context, the concept of functional food has been developed based on successive advances in the process of elaboration and understanding of the relationship between nutrition and health, considering that their purpose does not end with the mere satisfaction of the need for hunger but intend to provide the human body with essential nutrients to prevent nutrition-related diseases and even improve physical and mental health [5]. Therefore, foods that naturally bring health benefits should be distinguished from those modified, enriched and fortified to be defined as functional. In recent decades, this last category of food is the one on which research has focused following the development of a multitude of new foods and beverages and favoring the acquisition of a significant competitive advantage for companies owing to the demand for health foods has been stimulated by socio-economic changes, such as the lengthening of life expectancy, rising healthcare costs, the social costs of non-communicable diseases and the widespread desire for a better quality of life [6] and, because these foods can be distributed with nutritional and health claims, aiming to help consumers make well-informed choices at a glance [7,8,9], favoring a differentiation of foods that possess them.

Therefore, job roles, family organization, lifestyle and social changes [10] are making food intake more irregular, often without fixed mealtimes, leading to cardiometabolic consequences [11]. This irregularity can result in large time intervals between meals, while the human body requires a regular energy supply. Consumers often meet this energy need by eating snack products between meals, regularly outside the home and most frequently in the afternoon. As such, snacking becomes an important reward to consumers within modern eating behaviors [12].

**Figure 1:**
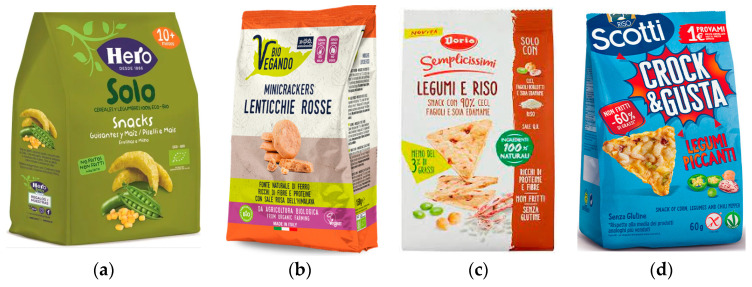
Samples of Italian ready-to-eat snacks. (**a**) Mix of cereals and legumes snack (on the label, we can observe the following main claims: organic, non-fried); (**b**) red lentils snack (on the label, we can observe the following main claims: bio, vegan, natural source of fibers, rich in proteins and fibers, with Himalaya red salt); (**c**) mix of legumes and rice snack (on the label, we can observe the following main claims: snack with 90% chickpeas, beams, edamame, less than 3% in fat, rich in proteins and fibers, non-fried, gluten-free); (**d**) mix of corn, legumes and chili pepper (on the label, we can observe the following main claims: hot legumes, gluten-free, non-fried, less than 60% in fat). Source: photos provided by the co-author Antonella Labbate, 2022.

Moreover, the consumption of snacks is motivated by biological factors (i.e., genetic profile, homeostasis), social factors (i.e., cultural, religion, education, beliefs, parental influences marketing, climate, food access, food availability), and hedonic factors (i.e., addiction, pleasure, sensory perceptions, emotions) [13,14,15,16]. In the market, there is a wide range of ready-to-eat (RTE) snacks on supermarket shelves with an extensive variety of sizes, shapes, colors and tastes designed to attract the consumer [Figure 1]. The major types of snacks are classified according to their ingredient composition and processing technology into first, second and third generation. The first category includes the simplest snacks such as chips, popcorn, etc. [17]; in the second category are those with direct expansion (puffed corn curls, onion rings, corn tortilla chips, etc.) which have a relatively low nutritional value [16]; finally, the third category includes snacks with indirect expansion, also called “pellets” which are first dried and then expanded by frying, hot air or microwave heating [18]. In general, the most used production techniques are baking, frying and extrusion.

### 1.2. Aims and Significance

**Figure 2:**
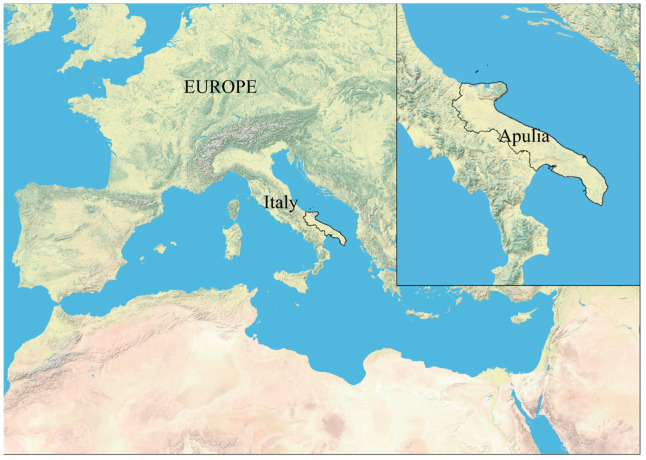
Geographical location on Apulia region (southeastern Italy).

In this context, the present paper had two major purposes. The first was to evaluate the label information (i.e., intrinsic: percentage of legumes, cooking method, presence and type of oil, use of organic ingredients, absence of gluten, etc., and extrinsic: packaging, claims, brand, etc.), as well as the place of the purchase in terms of their effect on a range of ready-to-eat snacks’ prices, offered by Italian stores and whereby consumers maximize their utility and products’ perceived quality. Additionally, this food category offered a range of innovation and healthiness which made it appropriate for the first purpose of this present research, for which we used the hedonic price model (HPM) that assumes that products consist of a set of intrinsic and extrinsic attributes valued by both manufacturers and consumers. In fact, a hedonic function represents the minimum price at which attributes can be given and the maximum price at which they will be purchased [19] in a long-run equilibrium. In parallel, the second purpose of this research was to explore, among a representative sample of Italian consumers in Apulia region (southeastern Italy, Figure 2; the total population and area are 4.06 million and 19.37 K km^2^, respectively) [20], determinants allowing to understand the behavior, preferences and purchasing decisions of Italian consumers towards ready-to-eat snacks based on legumes and, to estimate their willingness-to-pay (WTP) for such products, qualified as healthy and innovative food, produced in Italy and sold in the Apulian food stores. For this second purpose, we used the discrete choice experiment (DCE) approach, by means of a multinomial logit model (MNL) and latent class model (LCM), for which data were collected through a social survey. Scrupulously, this second purpose of the study addressed three interlinked research sections: (i) What are Apulian consumers’ habit and propensity towards consuming snack-based legumes? (ii) What is their WTP towards nutritional ingredients and labelling information for snack-based legumes? (iii) How does their socioeconomic profile affect their WTP? As such, this research contributes to the scientific literature through several features. First, there is a paucity of research that have used HPM for market analysis and/or DCE to investigate snack-based legumes consumers’ habits and preferences as supported by the literature review, presented above (Section 1.2). Second, the Italian snacking market has never been focused on consumer behavior, preferences, and WTP towards ready-to-eat snacks and, particularly, snack-based legumes. Moreover, this paper provides new indications into Italian snacking consumption in terms of preferences utility, whereby an average of 38 and 30 over 100 individuals snack at least a few times a week in Italia and Apulia, respectively [20]. Third, the market analysis through the hedonic price and the econometric valuation analysis is essential for the prediction and positioning of market and innovation outreach strategies [21] by snacks manufacturers that would meet Italian consumers’ expectations and, consequently, strengthen their competitiveness. Fourth, eliciting consumers’ habits and preferences also has public health implications in terms of increasing healthy eating early in life [22] and enhancing communication about nutritional ingredients and labelling claims on snacks to affect the healthy consumption of snacks products such snack-based legumes. In fact, a matching of the results obtained from the two models applied here (HPM and DCE) allow us to assess whether supply and demand are aligned, to understand if what is already on the store shelves corresponds to what is expected by the consumer and to guide the food industry in the development of product combinations more in line with market preferences. The following section embarks on how HPM and DCE were mapped out.

## 2. Materials and Methods

### 2.1. Hedonic Price Model (HPM)

#### 2.1.1. Theoretical Background

To estimate the monetary value of product features, the HPM, which has its roots in microeconomic theory, was first proposed by Rosen in 1974 [23]. HPM considers a product as a set of characteristics/attributes, whereby the consumers purchase the product that comprises this set, maximizing their utility. Similarly, producers maximize their profits by determining the price of a product based on its attributes [23]. Therefore, differentiated goods can be considered as a set of various quality attributes that discriminate them from other similar goods, so that the equilibrium market price can be considered a function of the implicit prices of each attribute of the good [24]. Nevertheless, the price function should not be directly interpreted as general measures of the consumer’s WTP for product attributes. In fact, a greater observed price for an attribute may be more influenced by the costs of production, rather than by the consumers’ appreciation. Moreover, there is a possibility that only a small fraction of consumers purchase goods that contain such expensive attributes. As such, HDM is generally recognized as a relevant tool for assessing the price premium for “credence attributes” such as certification, indications of origin and other characteristics that cannot be observed by the consumer after purchase [23,24], making it difficult to assess its utility. Given that, the price “P” of a product “j” can be described as:

Pj = f(Zj)(1)

where: “Z” is a vector of characteristics belonging to the product “j” and “f(.)” is an unspecified functional form.

Equation (1) indicates that the price (P) that consumers pay for the product is a function of the monetary values of a set of attributes (j) incorporated in the product (Z) sold on the market, which can be dimensioned by partially differentiating with respect to each attribute [23,25]. Furthermore, since at market equilibrium the marginal price that a consumer pays for each attribute j corresponds to the marginal cost that the producer incurs to offer that attribute on the market, Equation (1) can be estimated using ordinary least squares. Since variables with different scales can be selected, as well as dummy variables, it is necessary to find the optimal hedonic price model among the available forms, such as log-linear, double log-linear and linear log. In this study, a single equation approach was applied [26,27] to determine the effects that each snack characteristic causes on the price and a linear specification of the hedonic price equation were preferred. As such, the equation of the final model is given as follows:

P = β_0_ + β_j_ Z_i_ + β_j_ X_ij_ + ε (2)

where:

“P” is the price, “β_0_” is the constant, β_j_ is a parameter vector of product attributes, “Z” is the quantitative attribute “weight”, “X” is defined as a set of observable qualitative attributes of the product, “i” identifies an attribute (i = 1, ..., I), “j” is the number of choices between different qualitative attributes (j = 1, ..., J) and “ε” is residual.

#### 2.1.2. Implementation and Statistical Data Analysis

To apply Equation (2), we collected data between May 2022 and July 2022 on 177 valid observations on all legume-based snacks as presented on the shelves and sold in Apulia retail stores (i.e., hypermarkets, supermarkets, minimarkets and discounters) and e-shops. Among this information (Table 1), on the one hand, the retail price (“P”, ranging from EUR 0.73 to 3.53 per pack of 100 g) and the weight (“Z”, ranging from 40 to 240 g) were considered as dependent and continuous variables, while on the other hand the other explanatory variables “X” was categorical and then converted into one or more fictitious variables. Consequently, we estimated “P” by a mean of statistical linear regression, whereby we derived the values of “β_0_“and “β_j_”. Prior to the regression analysis, quantitative data (i.e., weight of the package) were normalized by Box-Cox power transformation to improve their conformity to normal distributions. The Shapiro–Wilk test was then used to test their normality.

### 2.2. Discrete Choice Experiment (DCE)

#### 2.2.1. Theoretical Background

The discrete choice experiment (DCE) constitutes a social survey-based approach, often used to estimate consumer preferences and behavior as well as their WTP. In line with Lancaster utility theory [28], DCE was introduced by Batsell and Lodish [29] and Louvière and Woodworth [30]. It simulates a purchase situation by presenting different products/alternatives to respondents and asking them to choose the product that, among all, best meets their requirements and expectations [21]. Generally, a constant alternative corresponding to non-buy is also presented to add more realism to the experiment. Concretely, a social survey-based approach is divided into sets of alternative possibilities consisting of at least two options and the no-buy choice (Table 2). The product is thus described by several attributes, further classified into levels. Consumers are invited to choose which product they would buy within each set of alternative options (choice sets) based on the description provided, revealing their preference for specific attributes or levels and their relative importance [31]. Thus, the consumer derives the marginal utility by examining the trade-off between the relevant attributes of a product to make certain purchasing decisions [32]. Over the years, the use of DCE has been extended to many disciplines, such as transport, environment, telecommunications, marketing and, recently, human health. Regarding agri-food products, the use of DCE has intensely increased in the last decade and has been applied for different themes such as, but not limited to, (i) wine consumption [21], (ii) enhancement of typical products [32], and (iii) local and organic foods [33].

#### 2.2.2. Implementation and Econometric Data Analysis

We implemented the DCE in 5 phases. First, we defined the attributes and related levels as depicted in Table 3 from the observational study during the survey conducted in the places of sale. Second, we determined the experimental design from the combination of nine selected attributes and their levels.

Consequently, we conducted a D-efficient Bayesian design [33,34,35], for which the D-error was low, equal to 0.214%, indicating a good efficiency of the design at extracting information from respondents in the DCE. Moreover, we used Equation (3), for which 4,145,760 possible profiles were created (i.e., J_n_ = 3^2^ × 5 × 2^6^, whereby two attributes have three levels; one attribute has five levels and six attributes present two levels as depicted in Table 3).

(3) $$N=\frac{\mathrm{Jn}\;\times \left(\mathrm{Jn}-1\right)}{2}$$

where: “J” corresponds to the number of levels and “n” to the number of attributes that have “J” levels.

As a result, we generated 48 reasonable profiles (i.e., set choices), consisting of 3 options (A, B and C), which were divided into two blocks consisting of eight set choices each (see Supplementary File S1—Experimental Design and Table 2 as an example), with a fixed order of presentation based on the previous experience of the focus group experts in Italian food consumption and preferences and the pilot survey. Each choice set consisted of 5 columns. The first two columns described the product A known as an option A, indicating a set of specific features available for consumers. The second two columns depicted the product B known as an option B, including another set of specific attributes of the legume snacks. The last column had no attributes and referred to option C, indicating the possibility of the “no-buy” of legume snacks that could be chosen by respondents. The use of photographs, as a stimulus visual information reflecting the attribute under assessment [36], helped them to easily choose the option.

Third, we carried out a field survey through an online questionnaire, from July to December 2022, reaching a valid sample of 518 respondents mainly in Apulia region, taking into consideration the Apulian population’s age, gender distribution and annual household income (Table 4).

As such, we organized the field questionnaire into three sections: the first section involved questions about the behavior and propensity to consume legume-based snacks in terms of general purchases, consumption and importance of legumes, knowledge and purchases of innovative legume snacks. Concretely, this section aimed to understand the level of knowledge of respondents of legume snacks. Specifically, we asked them to indicate: (i) the frequencies and places of purchase in general (i.e., neighborhood shops, neighborhood stores, hypermarkets, discounts, e-commerce), where they go most often to buy food products; (ii) the importance that they place with respect to certain product characteristics (i.e., label, brand, price, Bio, etc.), using a three-level staircase (i.e., not important, medium importance, very important); (iii) the type of diet, the type of snack and the number of meals during the day; (iv) the consumption and importance of legumes in the diet; and, (v) the knowledge, consumption and frequency of purchase of legume snacks. In the second section of the questionnaire, we engaged a hypothetical purchase situation of legume snacks, in which the blocks of eight sets of choice were presented to respondents as described above. Finally, in the third section, we collected the socio-economic and socio-demographic information of the respondents (i.e., age, income, residence, household income, etc.). Once data were collected, we based our econometric data analysis, using Nlogit software (version 5.0), on the Lancaster utility theory [28] as explored above. Here, we assumed that the consumer, by choosing between two products, A or B, will select the one with greater utility (Table 2). Therefore, the choice of the product is influenced by its attributes and the preferences that distinguish the consumers, conditioned by undetectable factors (for example habit or aversion), considered as random variables. As such, the utility function is described by a deterministic component (V), a function of observable attributes and a stochastic component (ɛ) that represents measurement errors with all unobservable attributes that influence the purchase choice. Thus, the utility function is determined as follows:

U_nj_ = V_nj_ + ɛ_nj_ (4)

where: V_nj_ can be expressed by:

V_nj_ = β’x_nj_ = α + β_1_x_1n_ + β_2_x_2n_ + ... + β_m_ x_mni_ + ɛ_nj_ (5)

“xnj” indicates the attribute “x” of the alternative “j” selected by the individual “n”, with the coefficients “β’”, whereby “β_n_” represents the weight of the preference for each level of attribute, while the coefficient “α” incorporates the heterogeneities of the sample of consumers/respondents.

Since it is not possible to know with exact certainty which alternative “j” is most useful for the respondent, it is determined that the alternative “j” is more useful than the other alternatives considered. Therefore, the probability that the respondent “i” will choose the alternative “j” among the others is expressed as follows:

(6) $$\begin{array}{cc} P_{\mathrm{ni}} & =\mathrm{Prob}\;\left(U_{\mathrm{ni}}>{\mathrm{In}}_{\mathrm{it}}\right)\;\forall_{j}\neq \mathrm{and}\\ & =\mathrm{Prob}\;({\mathrm{No}}_{}>\mathrm{In}\;\mathrm{nj}\;{+}_{000})\;\forall \neq i\\ & =\mathrm{Prob}\;{(}_{0000}<-{\mathrm{In}}_{\mathrm{nj}})\;\forall_{j}\neq i \end{array}$$

where: “n” is the single interviewee (i.e., consumer/respondent) and “j” is the alternative (i.e., Product A/Option A; Product B/Option B; No-buy/Option C as described above in Table 2).

Therefore, the overall average monetary value of each individual attribute reflects the “price premium” that the consumer would be willing to pay for a hypothetical product that possesses those characteristics. As such, we estimated this monetary value through a multinomial logit model (MNL) to which we applied an LCM to improve the likelihood of the model, segment the market and estimate a probable profile of the consumer of innovative snacks. Although the MNL provided the basis for the analysis of discrete choice modeling, its basic limitations, in particular the assumption of independence from irrelevant alternatives (IIA), motivated the researchers to consider specific alternatives, including the latent class model, as well as the approach used in this study to analyze the different behaviors among respondents. The LCM model allows us to group the consumers interviewed into a set of Q classes in relation to their purchasing choices. The initial sample is then divided into sub-samples, so that we no longer have a heterogeneous total sample, but a defined number of homogeneous sub-samples. Prior to selecting the most appropriate number of classes to improve the model statistical properties, the information criteria values for models with 1 to classes 4 has been elaborated (Table 5), in which a gradual process was conducted. As such, a model fit for statistics information criteria (IC: maximum log likelihood, minimum Bayesian information criteria/BIC and minimum Corrected Akaike Criteria/CAIC) is normally used for this purpose. By gradually adding the number of classes, the model presents the most optimum fit improvement in terms of stability, sensitivity and specificity [21,38,39]. Nevertheless, using AIC may risk overfitting the model by having too many classes and using BIC may risk underfitting the model by having too few classes [40]. Therefore, when determining the appropriate number of classes in an LCM, the researcher must consider the number of classes required to address the underlying research questions and the ease of interpretation of multiple classes when the number of classes is large [41]. For this reason, we decided to limit the LCM at two classes. The solution led to an improvement in the fit of the model compared to MNL, without incurring in a less stable convergence due to the increase in the class numbers.

Although the division of the sample through the LCM allows us to include from the beginning of the covariates, the characteristics of the respondents able to influence their purchasing behavior and optimize the subdivision based on socio-economic and/or physical peculiarities, given the exploratory nature of the survey it was decided to verify the significance of the covariates downstream of the segmentation. For this investigation, a decision-making process was carried out consisting of various test steps, with the aim of selecting several classes able to improve the statistical properties of the model considering the consistency of the classes in percentage terms, the significance attributes and the likelihood of the model. As a result of the selection of alternative j in the various purchase sets, the probability of consumer “i” to fall into class q is expressed by:

(7) $$\pi_{\mathrm{ij}\left|q\right.}=\frac{\exp\left(\beta_{q}^{'}x_{\mathrm{ij}}\right)}{\sum_{q=1}^{Q}\exp(\beta_{q}^{\prime }x_{\mathrm{ij}})}$$

where: “x_ij_” expresses a set of typical characteristics of the class; “β_q_” are specific coefficients relative to the classes to be estimated. The conditional probability that consumers will choose alternative “j” is expressed:

(8) $$\pi_{\mathrm{ij}}=\sum_{q=1}^{Q}\pi_{\mathrm{iq}}\pi_{\mathrm{ij}\left|q\right.}$$

To better explain the choices of consumers, the estimation of parameter values is carried out through the maximization of the log likelihood function:

(9) $$\mathrm{nL}=\sum_{i=1}^{N}\ln\left[\sum_{q=1}^{Q}\pi_{\mathrm{iq}}{\left(\prod_{t=1_{i}}^{T_{i}}\pi_{\mathrm{it}\left|q\right.}\right)}^{y_{\mathrm{ij}}}\right]$$

Regarding the estimation of WTP that reflects how much extra consumers are willing to pay as a price premium for a ready-to-eat legume-based snack with a specific characteristic, we used the following formula:

(10) $$\mathrm{WTPa}=-\frac{\beta a}{\beta p}$$

where: “WTPa” is the willingness-to-pay for attribute “a”; “β_a_” and “β_p_” are the estimated coefficients related to each attribute and price, respectively, according to the respondent’s membership in each class.

## 3. Results

### 3.1. Respondents’ Descriptive Statistics

This section comprises the main descriptive statistics related to the behavior and propensity of Apulian’s residents to purchase and consume legume-based snacks (i.e., part 1 of the field survey) and their socio-demographic and economic profiles (i.e., part 3 of the questionnaire). As such, supermarkets were the highest frequent purchase site for snacks (36.00%), followed by neighborhood shops (20.00%), hypermarkets (18.00%), grocery stores (16.00%), discount stores (10.00%) and E-commerce (1.00%), as shown in Table A3. Although around 53% of the respondents in this social survey accorded a high level of attention towards the expiry date of the products, followed by the type of ingredients, prices, nutrition facts and labels of snacks (Table A4). Regarding the type of snacks consumed among the sample (Table A5), few respondents (5.61%) selected the sandwich as a type of snack, but the majority (57.56%) of them opted for the fruits and vegetables (i.e., apple, banana, kiwi, peach, fennel, carrots, etc.). Furthermore, most respondents (45.95%) consumed legume snacks a few times a month, while few of them (3.47%) consumed this food category upon the recommendation of a dietitian, as presented in Table A6 and Table A7. Regarding their socio-demographic and economic profiles, on average, the respondents were similarly distributed between gender, since 48.80% were male, nearest to middle-age (46.12 years old), with an average of around 3 persons per family. In terms of academic level, the average number was almost 14 years of education. Furthermore, their total annual household income was distributed as follows: 57.10% (less than EUR 20,000), 29.00% (between EUR 20,000 and 40,000) and 13.90% (over EUR 40,000). Moreover, most of the respondents worked in the agriculture sector (16.22%), as depicted in Table 6.

### 3.2. Hedonic Price Results

#### 3.2.1. Descriptive Statistics and Analysis of Variance

Among the 177 valid observations (Table 7), 57.10% of snacks were organic certified. Chickpeas (65.00%) and corn (46.90%) were the most observed type of legumes and flour, respectively. Given the relative low observation of wheat flour (11.90%), most of the snacks were gluten-free (78.50%), not fried (61.00%), not spiced (87.00%) and formulated with sunflower oil (70.10%). In the same way, most of the items presented labels with at least one type of claim in order to enhance the snack consumption and encourage their potential purchase, such as: low fat (14.10%), source of proteins (65.00%) source of fiber (73.40%), vegan (25.40%). Additionally, most items (81.90%) indicated the recyclable symbol on their labels (Table 6). Moreover, snacks were widely observed in E-commerce (52.00%), followed by supermarkets (33.30%), neighborhood shops (5.60%), discount shops (5.60%) and hypermarkets (3.40%). Furthermore, the correlation between price (considered here as dependent variable) and these labels’ attributes (considered here as independent variables) revealed that the minimum price of a pack of legume snacks is equal to EUR 0.73 per 100 g, while the maximum price was EUR 3.53 per 100 g. Therefore, the analysis of variance depicted that the attributes with a highly significant difference (*p* < 0.001) were: “package size”, “lentils”, “rice”, “claim—low fat”, “claim—vegan”, “sunflower oil” and, “without oil”. Similarly, discount shops (*p* < 0.001) as a purchase site showed greater significance than other purchase places (Table 8).

#### 3.2.2. Linear Regression Analysis

The most frequently observed attributes were subsequently subjected to linear regression analysis. The empirical model showed a good overall significance (F = 47.005, *p* < 0.001) and a good ability to explain the variability of the dataset (R^2^ = 0.758), as shown in Table 8. Hence, the empirical model can now be expressed according to the following expression:

Price (EUR/100 g) = 120.660 + (−119.936 × “Package size”) + (0.277 × “Lentils”) + (0.340 × “Rice”) + (0.194 × “Potatoes”) + (0.133 × ”Claim Source of fibers”) + (−1.388 × “Claim Low fat”) + (0.187 × Claim Vegan) + (−0.124 × “Supermarket”) + (−0.503 × “Discount”) + (−0.580 × “Sunflower oil”) + (0.327 × “Without oil”).(11)

We considered 95 g per pack (an overall average of pack sizes) as a baseline value in the application of the hedonic price equation. Consequently, we assigned the values of 0 and 1 for variables presented in Table 7 and Table 8, respectively, in Equation (11). As a result, we obtained an average price of EUR 1.99 per 100 g. Subsequently, we calculated other prices this time by assigning a value of 1 to the variables that were significant through the previous descriptive analysis. Thus, on the one hand, the prices of snacks based on “lentils”, “rice flour”, “no oil”, “source of fibers”, “potatoes starch” and “vegan” were increased to EUR 2.26, 2.33, 2.31, 2.12, 2.18 and 2.17, respectively. On the other hand, the prices of legume-based snacks bought at the supermarket or at a “discount shop”, “with no fat” or “with sunflower oil” reduced to EUR 1.86, 1.48, 0.60 and 1.41, respectively, in comparison to the baseline price of EUR 1.99 per 100 g.

### 3.3. Econometric Results

#### 3.3.1. Multinomial Logit Model (MNL) and Latent Class Analysis (LCA)

To assess consumer preferences for the nine selected attributes as described above (Table 3), we initially used the MNL which provided the basis for the analysis of DCE. Then, we applied LCA that made it possible to group the interviewed respondents into a set of classes in relation to their purchasing choices. As such, the MNL estimates are reported in Table 9, and most coefficients of the concerned attributes (i.e., organic certification, gluten-free, indication of origin, recyclable package, the use of extra virgin oil) presented positive signs, except for price, chickpeas as a type of legume, the use of spices and sunflower oil, and were highly significant, except for chickpeas, at the significance level of 99%. These findings denote that the attribute with positive coefficients estimates and high significance provided Apulian consumers with high utility and trust regarding, at least, the quality of the legume-based snacks. On the contrary, the ASC—No choice coefficient (i.e., no buy) was equal to 0.076 and not significant, indicating that the thesis that respondents would gain utility from Product A or Product B over Option No-buy is not statistically supported. With respect to the LCA model, the sample was divided into 2 classes with values of 75.28% and 24.72%, improving the log likelihood function by indicating better values compared to the MNL (Table 9). Specifically, both classes have a highly significant and negative price coefficient, which means that, for the whole sample of respondents, a small price variation lead to not buying the product. On the contrary, the attributes related to the origin indication, the recyclability of the package and the use of an extra virgin olive oil were highly appreciated by the entire sample, since their coefficients had positive value, but with different significance levels among the two classes, accepting the hypothesis of heterogeneous consumer preferences for legume-based snacks consumption. Specifically, the purchase decision of the respondents of class 2 was not influenced, using chickpeas as a type of legumes, the addition of spices, the gluten claim and the use of sunflower as seed oil, for which their coefficients estimates were not significant. On the contrary, the correspondent coefficients with negative and significant levels in class 1 imply that the respondents of this class accorded a high degree of importance when selecting between products. With respect to the non-purchase variable, the correspondent coefficient was negative and highly significant for the first class, indicating that the respondents of this class were very inclined to purchase, while class 2 presented positive and significant coefficients, inducing a propensity not to buy, regardless of the type of attributes of the innovative legume-based snacks.

#### 3.3.2. Willingness-to-Pay

**Figure 3:**
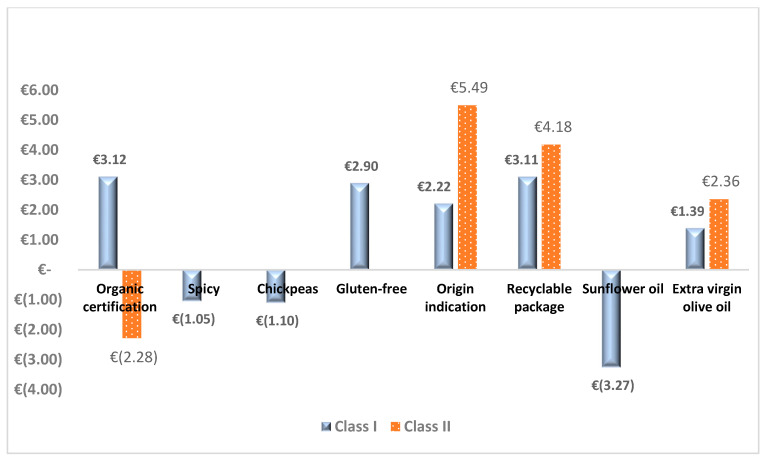
Willingness-to-pay for the use of nutritional ingredients and labelling claims on ready-to-eat legume-based snacks.

As Figure 3 depicts, the WTP of respondents for the use of nutritional and claims information on the label of legume-based snacks tends to be heterogeneous across the two classes. In class 2, consumers are willing to pay the highest price premium (EUR 5.49) for labelling regarding the regional origin of this food category, followed by class 1 (EUR 2.22). With respect to recyclable package claims and the use of extra virgin oil, all members’ classes are willing to pay a premium price range between EUR 3.11 and 4.18, and EUR 1.39 and 2.36, respectively. However, the WTP was negative for the use of spices, chickpeas as type of legumes and sunflower oil for members’ class 1. In addition, respondents’ from class 2 were not willing to pay a premium price for the organic certification of legume snacks. This can be explained by the fact that they do not have enough awareness about the beneficial aspect of biological products.

## 4. Discussion

### 4.1. Interpretation and Comparison of the Results

In this paper, ingredients, claims and certifications were the categories of determinants that may affect pricing and decisions on snacks purchase and consumptions by Italian to some extent. In terms of HDM, we found that not all intrinsic and extrinsic labelling information of the legume-based snacks were statistically significant. The presence of an organic certification, as widely observed, was considered not significant by the analysis of variance nor by the linear regression analysis. Moreover, on the one hand this factor and the peas as type of legumes did not influence the estimate of the hedonic price, while on the other hand, legume snacks based on lentils and chickpeas positively affected the hedonic price of the product. Above all determinants, the weight of the pack, slightly negative, presented a highly significant coefficient, indicating that larger packages would have a lower price (i.e., “family size” or “convenience format”). Similarly, the price was also negative and a highly significant coefficient. Moreover, the absence of oil and the presence of sunflower oil were considered as significant attributes but both negatively affect the estimate of the hedonic price, indicating that Italian consumers preferred or found greater utility from the presence of extra virgin olive oil and underlining the attention to eating health by the respondents. Furthermore, the substitution of wheat flour by rice flour and/or potato starch, traditionally gluten-free components and always used as substitutes for wheat flour in starchy foods and presenting positive and significant coefficients, was also greatly appreciated by Italian consumers. In addition, we found that there was a greater usefulness if lentils were added to the composition of the snack. On the contrary, the place of purchase (i.e., supermarkets and discount stores) presented negative coefficients, indicating a lower utility to the respondents. In terms of DCE, we found that the type of legumes, as a symbol of a healthy and eco-friendly product, was not so preferred by respondents, compared with its origin and certification. In fact, the results revealed that Italian consumers paid more attention to the presence of (i) an organic certification, (ii) an indication of origin and (iii) a claim of gluten-free, attributes that should be more considered by snack industries to continuously increase the utility of this food category.

Previous papers have also assumed the importance of food ingredients, claims and certifications in pricing and decisions on food consumption, such as snacks. In line with our HPM analysis, previous studies show that the global offer of legume-based snacks has strongly increased in recent years [42], many still focused on improving the technology of production of these highly innovative products [43,44,45,46], but very few have focused on the consumer and their propensity to buy. Given this research gap, the possibility of comparing our results with those emerging from other studies relating to the snack world has been reduced to a single possibility that investigates Ethiopian consumer preference and willingness to pay for enriched snacks [47]. Labeling, taste and ingredients were found to be among the most significant attributes in the process of choosing and purchasing innovative snacks, for both studies. In fact, in our study the respondents of the first class do not derive utility from a snack with a spicy flavor and the second class is indifferent to it. Ahmed et al. [47] found that Ethiopian consumers also prefer to buy mango flavored snacks rather than a spicy tomato one. The other important aspect that influences consumers’ decision to purchase snacks is the main ingredients: the sorghum-chickpea combination is much preferred over snacks with corn as the main ingredient, as consumers are aware that their blend has a better nutritional and/or health combination. So, this shows that consumers are more health conscious and choose protein-, fiber- and carbohydrate-enriched snacks over carbohydrate-enriched and protein-deficient snacks [47]. In the same way, our respondents are very attentive to the type of ingredients, nutritional indications and snack labeling, a constant that is also found in many other publications [48,49,50]. In fact, it is now established that consumers seek satisfaction in food, but at the same time they are increasingly aware of the fact that food contributes significantly to their health and well-being [51]. Health and well-being are sought not only in a careful evaluation of the main ingredients, but also of the minor constituents such as the type of oil used. In fact, the profiled consumers show a highly negative utility for the presence of sunflower oil (EUR −3.27 for the first class), as opposed to the willingness to pay a premium price for the presence of extra virgin oil (EUR 1.39 for first class and EUR 2.36 for the second class, respectively). Attention to health also emerges from the market analysis as, out of 100% of the observed products, 79% are gluten-free, 60% are not fried and each one has health and/or nutritional claims used by companies, both to highlight and enhance the beneficial effect of the product and because, in the wake of this trend, the consumer is more inclined to purchase. The importance of claims is seen both in the results of the DCE, which sees respondents more willing to pay for them, and in the study carried out in Ethiopia, which sees nutritional and/or health claims as the most influential attribute of fortified snacks. Although the presence of claims and the type of ingredients are attributes of ascertained importance for the consumer, to push companies to invest significantly in the promotion of these aspects, an even more influential attribute found to be significant in our study, is the presence of an indication of the origin of the raw materials constituting the snack. Although there is a different willingness to pay for this attribute, the purchase by both classes is strongly influenced by its presence, resulting in EUR 2.22 for the first class and EUR 5.49 for the second class. However, this interest is not reflected in the analysis of the hedonic price from which it emerges as only one Apulian producer (i.e., Cerealitalia) has presented on the label the indication of the geographical origin of the legumes used. Despite this lack of interest from companies, the literature appears to be in agreement with our result, showing how this attribute is already relevant for different types of products: plant-based beef meatballs [49], wine [21], sea urchins [52], oil [53] and legumes [54]. Depending on the social and cultural changes that have taken place, the needs and preferences expressed by consumers have changed, passing from the satisfaction of subjective needs and those related to the health of the individual, to ethical and environmental needs, paying attention to the community. Precisely in this context, attention to sustainability is growing, which takes the form of both the demand and the supply of innovative snacks with environmental claims on the label; in fact, most of the references observed on the shelf have on the label a reference to the green objectives pursued by the companies, ranging from the use of renewable energy sources to the use of recyclable packaging, attributes for which consumers of both classes have expressed a willingness to pay (EUR 3.11 for the first class and EUR 4.18 for the second class, respectively). The environmental aspects also include organic certification, which, as demonstrated by Juhl et al. [55] and Loureiro et al. [56], makes food to be perceived as healthier, more eco-friendly, and tastier than conventional ones. Aware of these reasons, most manufacturing companies require organic certification, which in fact has been found on a large percentage of products observed. However, not all respondents in our sample rate this attribute uniformly. In fact, if the first class is positively influenced by it and is willing to pay EUR 3.12, for the second class it is an attribute that discourages the purchase of the snack with a WTP of EUR −2.28. This result is in line with previous studies that have analyzed the same attribute on other agri-food products, underlining that the premium price that consumers are willing to pay for organic products compared to conventional ones does not follow a precise pattern [57]: WTP awards differ widely across countries, consumer segments and behavior, and product types for which consumers tend to be less loyal to processed organic foods than fresh ones [58]. Yiridoe et al. [59] found that consumers are willing to pay less for organic products with a longer shelf life. Uddin et al. [60] showed that consumers have a disincentive to purchase a certified organic ready-to-eat meal. So, one might think that if the first class is highly sensitive to the motivations that drive organic certification, the second class might not be sensitive to these aspects or, since it is a processed food, as in the cases mentioned above, it does not derive any usefulness from it.

### 4.2. Importance and Implications of the Results

The results explored above give a clear idea of the role of nutritional facts and labelling information of ready-to-eat legume-based snacks on Italian consumers’ habits, preferences and WTP. Here, we link the findings to the scientific literature and deduce private and public implications. Firstly, there is a dearth of research that has simultaneously implemented HPM and DCE to determine price and to elicit snacks consumers’ behaviors and their WTP, respectively. Hence, this study enriches the scientific literature on the elicitation of key determinants that affect legume snack price and choice by Italian consumers. Additionally, it may be considered as the starting point to concord a dialogue between the concerned industries interested in differentiating their food products and Italian consumers interested in increasing their consumption utility, and thus toward a better offer of innovative and healthy legume-based snacks. Among these snack products, potato chips often dominate the snack industry, followed by corn chips. Moreover, most RTE snacks are based on ingredients that are high in starch (i.e., corn, wheat, rice, oats, potatoes), with low nutritional value in terms of vitamins, minor minerals, amino acids, and fibers (Table A1) [61]. Furthermore, many RTE snacks are considered energy-dense and nutrient-poor foods with high glycemic index values (Table A2) [62], which could contribute to the increased prevalence of obesity and diabetes [63,64,65]. Consequently, over the last decade consumers turned out to be more health conscious and require healthier and more nutritious snacks than previously available. As such, the extruded legume-based snacks, in which legume proteins represent up to 20–35% [65] depending on the type of raw material, constitute an innovative healthier alternative to prevent coronary heart disease, diabetes and metabolic syndrome [66], and a suitable alternative for people intolerant to gluten [67]. This means that the launch of innovative and healthful products such a legume-based snack necessitates a campaign of promotion and communication through labelling, which are considered as an indispensable source of information for the consumer and, very often, the main tool by which the latter can evaluate the attributes of the product that they would not otherwise be able to maximize, such as its purchase utility and the product’s perceived quality [52]. On the one hand, these analyses have been widely applied to different products, such as lentils as such [54], processed tomatoes [68], ultra high temperature milk [27] and the yogurt market [26], but on the other hand, not yet enough attention has been paid to legume snacks, which are considered to be an innovative food that have been increasingly widespread in recent years.

### 4.3. Limitations and Future Research Directions

Despite being a representative sample of consumers of the Apulia population in terms of gender and ages, this research did not explore new insights into Italian regional differences in snack-based legumes preferences utility. Moreover, the DCE, as a non-incentive compatible design relying on hypothetical situations and stochastic assumptions, cannot predict all determinants affecting Italian consumers’ choice and WTP for ready-to-eat legume snacks. In addition, a randomized order of options in every choice set will be helpful to prevent an order bias and to ensure that the order in which options are presented to respondents will not influence the survey results. Therefore, we cannot definitively extrapolate our results to the overall Italian population in helping industries’ snacks to adopt market segmentation strategies at national level. As such, these results need to be validated by a further important research effort by also eliciting any other attributes (i.e., such as environmental sustainability, social sustainability, use of blockchain QR code as tool of traceability, etc.) attached to snack-based legumes and not considered in this research and in other reference markets other than Apulian.

## 5. Conclusions

Modernization and globalization have changed Italian consumers’ lifestyles and food choices. In some respects, ready-to-eat foods such snack-based legumes are progressively becoming more popular among them, owing to their convenience of consumption and ease of preparation and storage, inducing a substitution of the traditional meals to some extent. Hence, labeling information assists Italian consumers to purchase ready-to-eat snack-based legumes, for which they were willing to pay a premium price towards ingredients, nutritional claims, and certifications in terms of organic status and origin indication for adequate eating behavior. Henceforth, they mainly linked this food category to its nutritional benefits (i.e., high proteins and fiber contents, low fat, salt, and sugar content, gluten-free, rich in micronutrients and bioactive compounds) and its healthiest attributes (i.e., food alternatives that help to prevent coronary heart disease, diabetes, metabolic syndrome, etc.), but its ecological–environmental sustainability aspects (i.e., legumes as raw materials present favorable greenhouse gas emissions and water footprint compared with animal products, increase soil fertility and crop production by enriching the soil with the atmospheric nitrogen, decreasing the use of chemical fertilizers) remain partly known by consumers to some extent. This agro-ecological legumes feature may be relevant as an added value for snacks’ industries interested in promoting their products. From this viewpoint, the obtained results provide some insinuations in which to orient healthy and eco-friendly snack products’ enhancement policies and market strategies, based on consumer preferences.

## Figures and Tables

**Table 1:** Description of variables used in our hedonic price model analysis.

Variable	Description	Category
Price	Dependent variable Sales price EUR/pack Explanatory variable	Continuous
Package size	Package contents in grams	Continuous
Organic certification	Organic = 1; Non-organic = 0	Dichotomous
Type of legumes	Lentils = 1; Other = 0	Dichotomous
	Peas = 1; Other = 0	Dichotomous
	Chickpeas = 1; Other = 0	Dichotomous
Presence of other flours/starches	Corn = 1; Other = 0	Dichotomous
	Rice = 1; Other = 0	Dichotomous
	Potatoes = 1; Other = 0	Dichotomous
	Wheat = 1; Other = 0	Dichotomous
Gluten-free	Gluten-free = 1; Non gluten-free = 0	Dichotomous
Fried	Fried = 1; Not fried = 0	Dichotomous
Spices	Spicy = 1; Unspicy = 0	Dichotomous
Oil	Absent = 1; Other = 0	Dichotomous
	Extra Virgin Oil = 1; Other = 0	Dichotomous
	Seeds = 1; Other = 0	Dichotomous
Claim—low fat	Present = 1; Absent = 0	Dichotomous
Claim—source of proteins	Present = 1; Absent = 0	Dichotomous
Claim—source of fibers	Present = 1; Absent = 0	Dichotomous
Claim—vegan	Present = 1; Absent = 0	Dichotomous
Pack	Recyclable = 1; Non-recyclable = 0	Dichotomous
Observation site	Neighborhood shop = 1; Other = 0	Dichotomous
	Supermarket = 1; Other = 0	Dichotomous
	Hypermarket = 1; Other = 0	Dichotomous
	Discount = 1; Other = 0	Dichotomous
	E-commerce = 1; Other = 0	Dichotomous

**Table 2:** An example of a choice set used in our discrete choice experiment.

Option A (Product A)	Attribute	Option B (Product B)	Attribute	Option C (No-Buy)
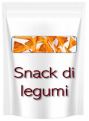	Certified organic (Bio)	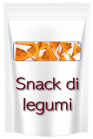	Not certified organic (Bio)	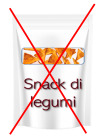
	Type of legumes:		Type of legumes:	
	Chickpeas 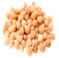		Lentils 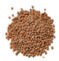	
	Gluten-free		Non gluten-free	
	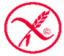		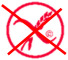	
	Presence of a nutritional claim		Absence of a nutritional claim	
	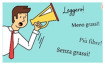		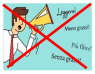	
	Spiced		Not spiced	
	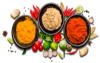		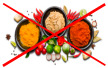	
	Presence of origin indication		Presence of origin indication	
	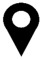		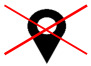	
	Recyclable package		Non-recyclable package	
	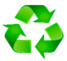		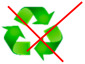	
	Type of oil: Sunflower		Type of oil: No added oil	
	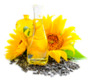		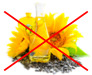	
	Weight		Weight	
	100 g		100 g	
	Price per pack		Price per pack	
	EUR 2.25		EUR 5.70	
I will select: ☑	Option A ☐		Option B ☐	Option C ☐

**Table 3:** Attributes and levels used in our discrete choice experiment.

Attribute of the Snack	Levels		Brief Description
Organic certification	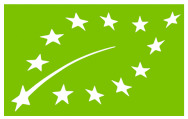	−1: Absence	An organic snack is produced without the use of synthetic chemicals and genetically modified organisms.
		+1: Presence	
Type of legumes	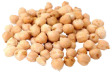	−1: Chickpeas	The type of legume that constitutes the main constituent of the snack.
	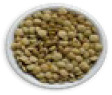	0: Lentils	
	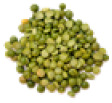	+1: Peas	
Gluten claim	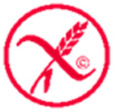	−1: Non gluten-free	Gluten is a cereal protein that causes intestinal inflammation for gluten intolerant consumers.
		+1: Gluten-free	
Nutritional or health claim	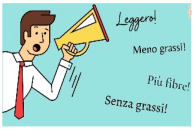	−1: Absence	The nutritional claim states, suggests or implies that the snack has beneficial nutritional properties, due to energy/substances contained. Similarly, the health claim states, suggests or implies the existence of a relationship between snack or one of its components and health.
		+1: Presence	
Spices	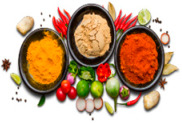	−1: Not spiced	The use or not of spices for flavouring or colouring snacks.
		+1: Spiced	
		-	
Origin indication	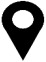	−1: Absence	The origin indicates the country or region in which snacks were produced.
		+1: Presence	
Recyclability of the pack	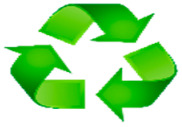	−1: Not recyclable	
		+1: Recyclable	
		-	
Type of oil	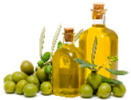	−1: Sunflower oil	The type of oil used or not as an ingredient.
		0: No added oil	
		+1: Extra virgin olive oil	
Price	−2: EUR 1.10 −1: EUR 2.25 0: EUR 3.40 +1: EUR 4.55 +2: EUR 5.70		Set of prices to make the hypothetical market more realistic with prices that respondents see daily in stores.

**Table 4:** Sampling of the respondents taking into consideration the age, gender and household income of the population in Apulia.

Variable	Category	Apulia	Sample	
		%	N.	%
Age (year)	From 19 to 30 Between 31 to 50 Over 50	17.00% 31.00% 52.00%	105 150 263	20.27% 28.96% 50.77%
Gender	Male Female	49.00% 51.00%	253 265	48.84% 51.16%
Annual household income (in EUR 1000)	Less than 20 Between 20 and 40 Over 40	Average 31,156	296 150 72	57.14% 28.96% 13.90%

Source: Istat, 2021 [37].

**Table 5:** The information criteria values for models 1 to 4 classes.

	Multinomial Logit	2-Class	3-Class	4-Class
Loglikelihood	−4340	−3792	−3620	−3483
K	10	21	32	43
Inf. CrAIC	8701	7226	7306	7053
AIC/K	2.10	1.84	1.76	1.70
BIC	−4257	−3617	−3353	−3125
N	4144	4144	4144	4144
Average classes probabilities	100%	75.3% 24.7%	64.3% 20.9% 14.7%	57.5% 14.5% 13.3% 14.6%

**Table 6:** Respondents’ socio-demographic and economic profiles.

Variable	Category	N.	Mean/%	Standard Deviation	Min	Max
Gender	Female	253	51.20%			
	Male	265	48.80%			
Age	Years		46.12	15.22	18	75
Family numbers	Number		3.19	1.12	1	5
Academic level	Years		13.94	3.85	0	21
Annual household income (in EUR 1000)	Less than 20	296	57.10%			
	Between 20 and 40	150	29.00%			
	Over 40	72	13.90%			
Sector of work	Agriculture	84	16.22%			
	Construction	34	6.56%			
	Culture and Art	21	4.05%			
	Finance	24	4.63%			
	Education	53	10.23%			
	Consultancy	58	11.20%			
	Industry and Transport	20	3.86%			
	Marketing and Communication	34	6.56%			
	Social Health	58	11.20%			
	Public Sector	18	3.47%			
	Tourism	40	7.72%			
	Students/not working	74	14.29%			

**Table 7:** Descriptive statistics of snacks used in our hedonic price model analysis.

Variable	Description	Number of Observations (Snack)	%	Mean	Standard Deviation	Min	Max
Package size	Package contents in grams	177		95.06	45.61	40.00	200.00
Price	EUR per 100 g	177		2.08	0.62	0.73	3.53
Organic certification	Organic	177	57.10				
Type of legumes	Lentils	177	59.90				
	Peas	177	58.20				
	Chickpeas	177	65.00				
Presence of other flours/starches	Corn	177	46.90				
	Rice	177	33.90				
	Potatoes	177	17.50				
	Wheat	177	11.90				
Gluten-free	Gluten-free	177	78.50				
Fried	Fried	177	39.00				
Spices	Spicy	177	13.00				
Oil	Absent	177	21.50				
	Extra Virgin Oil	177	14.70				
	Sunflower	177	70.10				
Claim—low fat	Present	177	14.10				
Claim—source of proteins	Present	177	65.00				
Claim—source of fibers	Present	177	73.40				
Claim—vegan	Present	177	25.40				
Pack	Recyclable	177	81.90				
Observation site	Neighborhood shop	177	5.60				
	Supermarket	177	33.30				
	Hypermarket	177	3.40				
	Discount	177	5.60				
	E-commerce	177	52.00				

**Table 8:** Parameter estimates in our hedonic price model analysis.

Variable	Coefficient (b)	Significance	*p*-Value
Constant	120.660	***	<0.001
Package size (g)	−119.936	***	<0.001
Lentils	0.277	***	<0.001
Rice	0.340	***	<0.001
Potatoes	0.194	**	0.019
Claim—source of fibers	0.133	**	0.019
Claim—low fat	−1.388	***	<0.001
Claim—vegan	0.187	***	0.003
Sunflower oil	−0.580	***	<0.001
Without oil	0.327	***	0.010
Supermarket	−0.124	**	0.031
Discount	−0.503	***	<0.001
R^2^	0.758		
Adjusted R^2^	0.742		
F-statistic	47.005		

Note: ***, ** => Significance at 99%, 95%, level, respectively.

**Table 9:** Multinomial logit model (MNL) and latent class analysis (LCA) results.

Multinomial Logit Model (MNL)			Latent Class Analysis (LCA)			
			Class 1		Class 2	
100%			75.28%		24.72%	
Attribute	Coefficients					
	Coefficient	*p*-value	Coefficient	*p*-value	Coefficient	*p*-value
Price	−0.13587 ***	0.000	−0.17553 ***	0.000	−0.14965 **	0.0248
Organic certification	0.42048 ***	0.000	0.55767 ***	0.000	−0.37660 **	0.0312
Type of legumes—chickpeas	−0.06728	0.268	−0.19830 ***	0.0066	0.18002	0.3411
Gluten claim—gluten-free	0.33102 ***	0.000	0.51665 ***	0.000	−0.22098	0.2116
Spices—spiced	−0.16627 ***	0.0002	−0.18565 ***	0.0002	−0.03238	0.865
Origin indication	0.35148 ***	0.000	0.38232 ***	0.000	0.84242 ***	0.000
Recyclability of the pack—recyclable	0.44088 ***	0.000	0.54381 ***	0.000	0.63196 ***	0.0006
Type of oil—sunflower	−0.42070 ***	0.000	−0.57562 ***	0.000	−0.30223	0.1393
Type of oil—extra virgin olive oil	0.29699 ***	0.000	0.24193 ***	0.0013	0.35899 *	0.0884
Opt-out (no choice)	0.07669	0.435	−0.96220 ***	0.000	2.22685 ***	0.000
**Model statistics**						
	MNL		LCA			
Log Likelihood (LL)	−4340.48		−3792.06			
Inf.Cr.AIC	8701.0		7626			
AIC/N	2.10		1.84			
Bayesian information criterion (BIC)	−4257		−3617			
Number of observations	4144		4144			
Total number of responses	518		518			
Number of variables (K)	10		21			

Note: ***, **, * ==> Significance at 99%, 95%, 90% level, respectively.

## Data Availability

Data are available from the corresponding author.

## Supplementary Materials

The following supporting information can be downloaded at: https://www.mdpi.com/article/10.3390/nu15071799/s1, Table S1: Experiment Design.

## Appendix A

**Table A1:** Composition of selected ready-to-eat snacks.

	Starch (g/100 g)	Sugar (g/100 g)	Dietary Fiber (g/100 g)	Fat (g/100 g)	Protein (g/100 g)	Water (g/100 g)	Energy Values (Kcal/100 g)
Popcorn	15.5	62.1	4.5	20	2.1	2.6	480
Potato crisps	52.6	0.7	5.3	34.2	6.2	2.8	530
Tortilla chips	58.9	1.2	6	22.6	7.6	0.9	459
Breadstick	67.5	5	3.8	8.4	11.2	3.5	392
Cereal bar	28.3	27.6	4.8	22.2	10.4	2.6	468
Kit Kat	12.9	50.1	1.4	26	7.5	2	500
Crispbread	67.4	3.2	11.7	0.6	9.4	6.4	308
Rice Krispie	82.5	10.4	0.7	1	6.1	3	382
Corn Flakes	81.4	8.2	0.6	0,9	7.9	3	376

Source: Retrieved from Public Health England [61].

**Table A2:** Glycemic index values of selected ready-to-eat snacks.

	Glycemic Index (Glucose = 100)	Glycemic Index (Bread = 100)
All Bran	30	43
Bagel	72	103
Breakfast cereal bar	78	111
Cheerios	74	106
Corn chips	63	90
Cupcake	73	104
Mars bar	68	97
Pretzel	83	119
Puffed crisp bread	81	116
Puffed rice cake	91	128

Source: Elaborated from Foster-Powell and Brand-Miller [62].

**Table A3:** Purchase sites by frequency of food purchase (in % of respondents).

Purchase Site		Frequency (%)	
	Low	Medium	High
Grocery stores	21.00%	22.00%	16.00%
Neighborhood shops	15.00%	19.00%	20.00%
Supermarkets	51.00%	39.00%	36.00%
Hypermarkets	6.00%	2.00%	18.00%
Discount stores	6.00%	18.00%	10.00%
E-commerce	0.00%	0.00%	1.00%

**Table A4:** Self-level attention on the features of food among the sample (in % of respondents).

Features of Food	Number	%
Price	224	43.20%
Label	190	36.70%
Nutrition facts	208	40.20%
Type of ingredients	238	45.90%
Quality of ingredients	167	32.20%
Packaging	94	18.10%
Gluten-free	34	6.63%
Indication of the product origin	143	27.65%
Organic food	58	11.22%
Expiry date	275	53.10%

**Table A5:** Type of snacks consumed among the sample (in % of respondents).

Type of Snack	Number	%
Fruits and vegetables (i.e., apple, banana, kiwi, peach, fennel, carrots, etc.)	298	57.65%
Salt snack (i.e., chips, crackers, gall salty biscuits)	222	42.86%
Sweet (i.e., croissants, chocolat cake, tarte, etc.)	150	29.08%
Sweet snack (i.e., wafers, biscuits, chocolates, etc.)	116	22.45%
Energy bars (i.e., protein, chocolate, red fruits, etc.)	68	13.27%
Sandwich	29	5.61%
Yogurt	142	27.55%
Other	23	4.59%

**Table A6:** Frequency of consumption of legume-based snack (in % of respondents).

Frequency of Consumption of Legume Snacks	N.	%
Never	137	26.45%
Everyday	3	0.58%
Several times a week	48	9.27%
Several times a month	55	10.62%
Few times a month	238	45.95%
Once a week	37	7.14%

**Table A7:** Reason of purchase of legume-based snack (in % of respondents).

Reason for Purchase	n.	%
Flavour	177	34.17%
Healthy	332	64.09%
Upon a recommendation	71	13.71%
Upon the advice of the dietitian	18	3.47%
